# Maggot Instructor: Semi-Automated Analysis of Learning and Memory in *Drosophila* Larvae

**DOI:** 10.3389/fpsyg.2018.01010

**Published:** 2018-06-20

**Authors:** Urte Tomasiunaite, Annekathrin Widmann, Andreas S. Thum

**Affiliations:** ^1^Department of Biology, University of Konstanz, Konstanz, Germany; ^2^Department of Molecular Neurobiology of Behavior, Georg-August-University Göttingen, Göttingen, Germany; ^3^Department of Genetics, University of Leipzig, Leipzig, Germany

**Keywords:** *Drosophila* larvae, aversive olfactory conditioning, optogenetics, olfactory receptor neurons, electric shock, mushroom body

## Abstract

For several decades, *Drosophila* has been widely used as a suitable model organism to study the fundamental processes of associative olfactory learning and memory. More recently, this condition also became true for the *Drosophila* larva, which has become a focus for learning and memory studies based on a number of technical advances in the field of anatomical, molecular, and neuronal analyses. The ongoing efforts should be mentioned to reconstruct the complete connectome of the larval brain featuring a total of about 10,000 neurons and the development of neurogenic tools that allow individual manipulation of each neuron. By contrast, standardized behavioral assays that are commonly used to analyze learning and memory in *Drosophila* larvae exhibit no such technical development. Most commonly, a simple assay with Petri dishes and odor containers is used; in this method, the animals must be manually transferred in several steps. The behavioral approach is therefore labor-intensive and limits the capacity to conduct large-scale genetic screenings in small laboratories. To circumvent these limitations, we introduce a training device called the Maggot Instructor. This device allows automatic training up to 10 groups of larvae in parallel. To achieve such goal, we used fully automated, computer-controlled optogenetic activation of single olfactory neurons in combination with the application of electric shocks. We showed that *Drosophila* larvae trained with the Maggot Instructor establish an odor-specific memory, which is independent of handling and non-associative effects. The Maggot Instructor will allow to investigate the large collections of genetically modified larvae in a short period and with minimal human resources. Therefore, the Maggot Instructor should be able to help extensive behavioral experiments in *Drosophila* larvae to keep up with the current technical advancements. In the longer term, this condition will lead to a better understanding of how learning and memory are organized at the cellular, synaptic, and molecular levels in *Drosophila* larvae.

## Introduction

Various technical and conceptual successes have helped recent research to gradually understand how a brain organizes learning and memory. Although, we still cannot understand and address a number of basic mechanisms, recent achievements are fascinating. Part of this development is due to the work on less complex insect brains, such as that of the fruit fly *Drosophila* and its larva (Heisenberg, 2003; Gerber and Stocker, 2007; Gerber et al., 2009; Busto et al., 2010; Diegelmann et al., 2013; Waddell, 2013, 2016; Cognigni et al., 2017; Widmann et al., 2017).

The benefits that the *Drosophila* larva offers for the analysis of learning and memory are based on several factors. First, the elementary organization of the larval central nervous system consists of only about 10,000 neurons (Dumstrei et al., 2003; Nassif et al., 2003). Second, the availability and robustness of behavioral assays that also allow to specifically address distinct memory phases (Aceves-Pina and Quinn, 1979; Scherer et al., 2003; Widmann et al., 2016). Third, the existence of transgenic techniques, which allow manipulation of neuronal networks, small sets of neurons, or even individually identified neurons (Luan et al., 2006; Pfeiffer et al., 2010; Li et al., 2014). Especially the establishment of a large set of single-cell split-Gal4 lines specific for the larval memory center – the mushroom body (MB) – has to be highlighted (Saumweber et al., 2018). Finally, the establishment of the larval connectome that includes the reconstruction of every individual neuron with all its synapses and synaptic partners (Ohyama et al., 2015; Berck et al., 2016; Jovanic et al., 2016; Schlegel et al., 2016; Eichler et al., 2017). These advantages now allow, for the first time, projects that can purposefully investigate – by using thousands of newly established genetic tools – how learning and memory are organized at the level of the brain, the nerve cell and the synapse.

The study of large amounts of different transgenic animals is simplified by the use of automated methods for behavioral research. However, in contrast to the adult *Drosophila*, these techniques are unavailable for the analysis of learning and memory in larvae (Colomb et al., 2009; Schnaitmann et al., 2010; Aso and Rubin, 2016; Ichinose and Tanimoto, 2016). The majority of behavioral learning assays in use are based on the principle of classical conditioning (aka Pavlovian conditioning) (Pavlov, 1927). In such studies, a biologically active stimulus (e.g., appetitive stimulus: food; aversive stimulus: electric shock), the unconditioned stimulus (US), is paired with a previously neutral stimulus (e.g., an odor), the conditioned stimulus (CS).

For almost 40 years (Aceves-Pina and Quinn, 1979), standard assays have been used on agar or agarose-filled Petri dishes and are very robust, easy to learn, inexpensive and require no complex technology (Gerber and Stocker, 2007). At the same time, however, such assays are time-consuming and labor-intensive, as the larvae have to be manually transferred to different Petri dishes during the entire experiment. In total, depending on the applied training regime, the conditioning of one group of animals using standard assays requires an average of 45–60 min. Consequently, this condition makes standard assays suitable to a limited extent for use in large behavioral screens. However, given the establishment of thousands of different genetic tools manipulating precisely the larval brain at the cellular and molecular level, such screens are becoming more important (Li et al., 2014; Saumweber et al., 2018). To use these resources extensively for larval learning and memory research, behavioral experiments or at least parts of them should be automated.

Thus, we designed the Maggot Instructor, a device to train *Drosophila* larvae in an automated fashion. The applied behavioral protocol uses electric shock as US paired with the artificial activation of a single olfactory receptor neuron (ORN) as CS (instead of a real odor). *Drosophila* larvae receive olfactory stimuli via the dorsal organ, a single sensillum located on the right and left sides of the head, with each housing 21 ORNs (Singh and Singh, 1984; Oppliger et al., 2000; Fishilevich et al., 2005; Kreher et al., 2005). For a specific odor, the dedicated ORNs or combinations of ORNs perceive the respective sensory information and signal it further to the larval main olfactory center – the antennal lobe (AL) (Fishilevich et al., 2005; Kreher et al., 2005; Ramaekers et al., 2005). All ORNs connect directly in a one-to-one fashion to 21 uniglomerular projection neurons (PNs). Most of the uniglomerular PNs in turn are directly connected to single-claw Kenyon cells (KC) in the MB calyx region (Eichler et al., 2017). Therefore, for almost every input channel, a direct connection from an ORN (first order) to a PN (second order) to a KC (third order) exists. As a consequence, optogenetically, individual ORN input channels can be activated to generate odor-specific learning and memory in the MB via simultaneous application of a US (Honda et al., 2014). However, in addition to this labeled line pathway, 14 additional multiglomerular PNs exists and initially about 100 KCs (in young L1 larvae) are randomly associated to two or more PNs (Berck et al., 2016; Eichler et al., 2017). These neurons can process odor information at different levels in a more integrative fashion.

To artificially activate the defined neurons, sophisticated optogenetic methods, which benefit from the semitransparent cuticle of the larvae, have been introduced (Schroll et al., 2006; Dawydow et al., 2014; Rohwedder et al., 2015). By using a two-part expression system, such as the Gal4/UAS system (Brand and Perrimon, 1993), proteins like channelrhodopsin2 (ChR2) or its improved variant ChR2-XXL (Schroll et al., 2006; Dawydow et al., 2014), a light-activated cation channel, can be possibly expressed to depolarize neurons by blue light in a time-wise precisely controlled manner. Single-cell specificity for ORNs can be achieved by using an established set of Or-Gal4 lines that use different Or promoter gene fragments to direct Gal4 expression to individual neurons (Fishilevich et al., 2005). Double-activation learning and memory experiments also become possible by replacing sugar reward (the US) by thermogenetic activation of octopaminergic (OA) neurons with the dTrpA1 channel and odor stimuli (the CS) by optically activating an ORN with ChR2 (Honda et al., 2014). This experiment is feasible as OA and dopamine (DA) neurons mediate sugar reward information in the larval brain (Selcho et al., 2014; Rohwedder et al., 2016; Saumweber et al., 2018). By contrast, the perception of electric shock by the *Drosophila* larva remains unelucidated. However, the DA system is also sufficient and necessary for aversive olfactory learning and memory in the larvae (Selcho et al., 2009). Four DA neurons innervating the vertical lobe, the lateral appendix, and the lower peduncle of the MB are possibly crucial for signaling aversive stimuli (Eichler et al., 2017).

The current model suggests that during training, a certain pattern of KCs activated by an odor (or in our case by artificial activation by light) occurs simultaneously with a modulatory signal about the aversive or appetitive US mediated by different sets of DA neurons (Heisenberg, 2003; Waddell, 2013, 2016). Coincident activation of KCs will in turn change the synaptic connectivity of KCs onto extrinsic MB output neurons (MBONs). Thus, during learning, MBONs change their response properties and act as odor-specific neurons that report the presence of a particular odor as an alerting signal for the conditioned behavior. The Maggot Instructor automates this step by executing the behavioral training protocol independently in a high-throughput manner.

## Materials and Methods

### Fly Stocks (Keeping and Crossing)

Fly strains were reared on standard *Drosophila* medium at 25°C in complete darkness. Or42b-Gal4 (Bloomington Stock No: 9972), Or47a-Gal4 (Bloomington Stock No: 9982), UAS-ChR2-XXL (Bloomington Stock No: 58374) and w^1118^ (obtained from R. Stocker) were used. Strains crossed with w^1118^ served as controls. For all the behavioral experiments, the flies were transferred to new vials and allowed to lay eggs for 2 days. Third instar feeding-stage larvae aged 96–144 h were used for behavioral experiments.

### Assay Plates and Odors

Petri dishes (85 mm diameter; Cat. No. 82.1472, Sarstedt, Nümbrecht) were used as the test plates, as described previously (Pauls et al., 2010b; Huser et al., 2012, 2017; Gerber et al., 2013). The test plates and training chambers were filled with 2.5% agarose (Sigma-Aldrich, Cat. No. A9539, CAS No. 9012-36-6). In several behavioral experiments 0.01 M lithium chloride (Sigma-Aldrich, Cat. No. 298328, CAS No. 85144-11-2) was mixed with 2.5% agarose. Throughout the test, the Petri dishes were covered with perforated lids for an equal distribution of odors. All the experiments were performed at about 21°C. As olfactory stimuli in the test we used 10 μl amyl acetate (AM, Sigma-Aldrich, Cat. No. 46022; CAS No. 628-63-7; diluted at 1:100, 1:250, 1:500, 1:600 and 1:750 in paraffin oil, Sigma-Aldrich, Cat. No. 76235, CAS No. 8012-95-1), benzaldehyde (BA, undiluted; Sigma-Aldrich, Cat. No. 12010, CAS No. 100-52-7) and ethyl acetate (EA, Sigma-Aldrich, Cat. No. 270989; CAS No. 141-78-6; diluted 1:1000 in paraffin oil, Sigma-Aldrich, Cat. No. 76235, CAS No. 8012-95-1). Odorants were loaded into custom-made Teflon containers (4.5-mm diameter) with perforated lids (Scherer et al., 2003) and were used for no longer than 5 h after preparation.

### Experimental Setup/Compact Real-Time Input Output (cRIO)

The Maggot Instructor consists of a training box wired with a computer that controls the type and timing of the applied stimuli via a cRIO system and an automated training device (ATD) (Graetzel et al., 2010; Kain et al., 2012; Dylla et al., 2017). cRIO (NI 9074) from *National Instruments* was used as a controlling device for the automated training protocol. cRIO was also used to regulate and monitor the technical aspects, such as the fine adjustment of parameters (e.g., light intensity, voltage, or temperature). The software *Build Digital Output Sequence with Frequency Output (BDOS)* was used for programming cRIO (Dylla et al., 2017). All settings in cRIO were transmitted to the training box (see below), where the parameters, including electric shock or light intensity were adjusted appropriately. Larval training was carried out in an elongated metal box (the training box), which was separated into 10 training chambers with the same size and can be regulated in parallel or individually. Each chamber consists of a case with an electrode at the front and rear end, a Peltier element underneath the chamber and odor inlets and outlets on all four sides. The training chamber is closed by a lid, which contains a white and a blue LED.

### Training Protocol

Only L3 larvae that are in the feeding stage were used. This requirement was achieved by collecting the larvae from the top layer only of the food substrate. Ten groups with 30 larvae each were collected, washed with tap water, and stored in a water drop for up to 30 min before the experiment. To avoid artificial activation of ORNs in the experimental animals, these steps were performed under red light. Before the experiment, the training chambers were filled with 2.5% agarose to cover the entire bottom with a substrate layer of about 1 cm thickness. After the preparation, the larvae were transferred to the training chambers. The larvae from every genotype were used in each run. For several runs, the training chambers were consistently varied for each genotype. Several runs were possible per training chamber with the same agarose substrate. To prevent the larvae from escaping the training chambers, a custom-made plastic frame covered with a plastic net was inserted into each training chamber. This technique was established by Khurana et al. (2009). This method also prevented the larvae from climbing the training chamber and thus avoiding electric shock. The training chambers were also moistened with about 1 ml of tap water to ensure the proper hydration of the larvae. Afterward, the lids of each training chamber and the cover of the Maggot Instructor were closed. The device was switched on, and the previously defined training protocol was started. All the training steps including CS (if not otherwise mentioned at a light intensity of about 86,000 lux) and US (if not otherwise mentioned electric shock of 120 V) application, then ran automatically. The training lasted for 60 min.

After training, the cover of the Maggot Instructor and the lids of each training chamber were removed. For the test, the larvae from each training chamber were placed on a fresh, pure agarose assay plate with an odor container on the one side and a second container without olfactory cue on the other side. The sides were randomly changed for every training chamber. All the larvae from one training chamber located on the plastic frames and the agarose cover bottom were collected and transferred. The larvae were placed in the center of the Petri dish, the lid was closed, and the larvae were given 5 min to freely move on the test plate. Ten test plates were analyzed in parallel (one for each training chamber). A Preference Index was calculated by subtracting the number of larvae on the control container side (CC) from the number of larvae on the odor side (*ODOR*) and dividing the result by the total number of larvae on both sides and in the middle zone (TOTAL):

Preference Index=(#ODOR−#CC)/#TOTAL

The positive values indicate attraction to the odor, whereas the negative values represent aversion.

### Statistical Analysis

All data processing, statistical analyses, and visualizations were conducted with GraphPad Prism 7.0a. Figure alignments were performed with Adobe Photoshop CC. The groups that showed no violation of the assumption of normal distribution (Shapiro–Wilk test) and homogeneity of variance (Bartlett’s test) were analyzed with parametric statistics. One-way ANOVA was applied followed by planned pairwise comparisons between the relevant groups with a Tukey’s honestly significant difference *post hoc* test (comparisons between groups larger than two). Experiments with data that significantly differed from the assumptions above were analyzed with the non-parametric Kruskal–Wallis test followed by Dunn’s multiple pairwise comparison. To compare single genotypes against chance level, we used one sample *t*-test or Wilcoxon signed-rank test. The significance level of statistical tests was set to 0.05. Data were presented as box plots, with 50% of the values of a given genotype being located within the boxes and the whiskers representing the entire set of data. Outsiders are indicated as dots. The median performance index was indicated as a bold line and the mean as a cross within the box plot.

## Results

### Maggot Instructor: A Custom-Made, Automated Approach to Train Larvae

**FIGURE 1:**
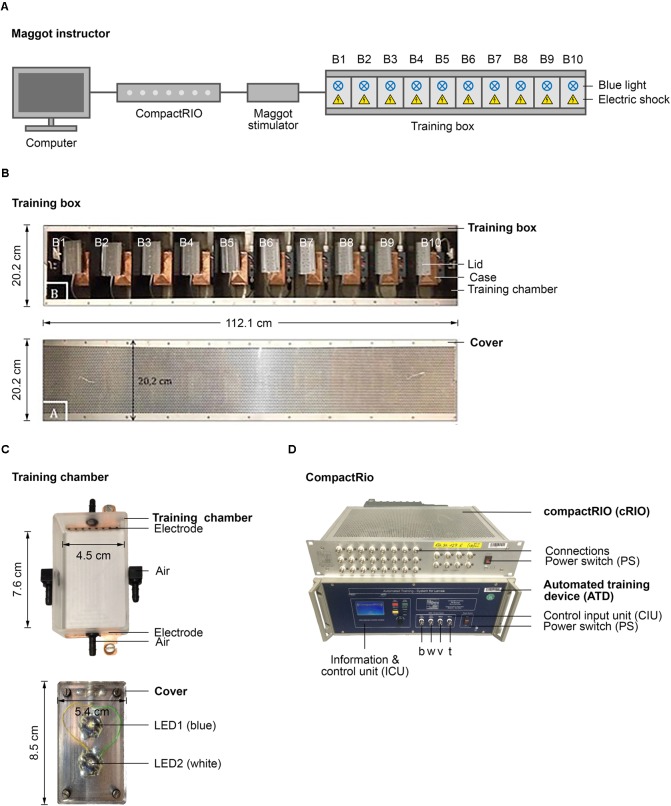
Semi-automated conditioning device. **(A)** Schematic overview of the Maggot Instructor. Setup consists of a computer, compactRIO (compact Real-time Input Output), maggot stimulator and a training box. The training box is split in ten training chambers to parallelize larval training. Each training chamber has a source of light and electric shock. **(B)** Shows the training box on top and its cover at the bottom. **(C)** Shows a training chamber at the top and its lid that includes two LEDs at the bottom. **(D)** Shows the compactRIO system and the connected custom-made automated training device. b, w, v, and t show the connections for the blue and white light, the voltage channel and the temperature channel, respectively.

A comprehensive set of standardized behavioral assays is available to analyze learning and memory in *Drosophila* larvae (Gerber and Stocker, 2007; Widmann et al., 2017). These approaches all require the larvae to be transferred manually several times from one Petri dish to another during the procedure and are thus labor intensive. To overcome this limitation, we aimed to develop a new, robust, and easy-to-handle device, which we named Maggot Instructor, to train *Drosophila* larvae in an automated fashion. The device consists of a training box connected to a computer that controls the type and timing of the applied stimuli via a cRIO system and an ATD (**Figures 1A,D**) (Dylla et al., 2017). Both are programmed by simple and flexible customizable training protocols using a BDOS software (Dylla et al., 2017). The training box consists of 10 separate training chambers that can be regulated in parallel or individually (**Figures 1A,B**). Therefore, one can train up to 10 groups of larvae in this device in parallel to increase the throughput. Each training chamber consists of a case, in which an electrode is incorporated at the front and the rear end (**Figure 1C**, above). In addition, a Peltier element is placed underneath the chamber and the odor inlets and outlets on all four sides (**Figure 1C**, above). The training chamber is closed at the top by a lid equipped with a white and a blue LED (**Figure 1C**, below). Therefore, the larvae can be exposed to the following stimuli: cold, heat, air, electric shock, and light (white and blue). Additional technical details are included in **Figure 1**, in Section “Materials and Methods,” or are available upon request. Our initial study focused on a protocol that automatically conditions the larvae by optogenetic activation of ORNs (CS) via blue light and stimulation through electric shock (US).

### Training Procedure

**FIGURE 2:**
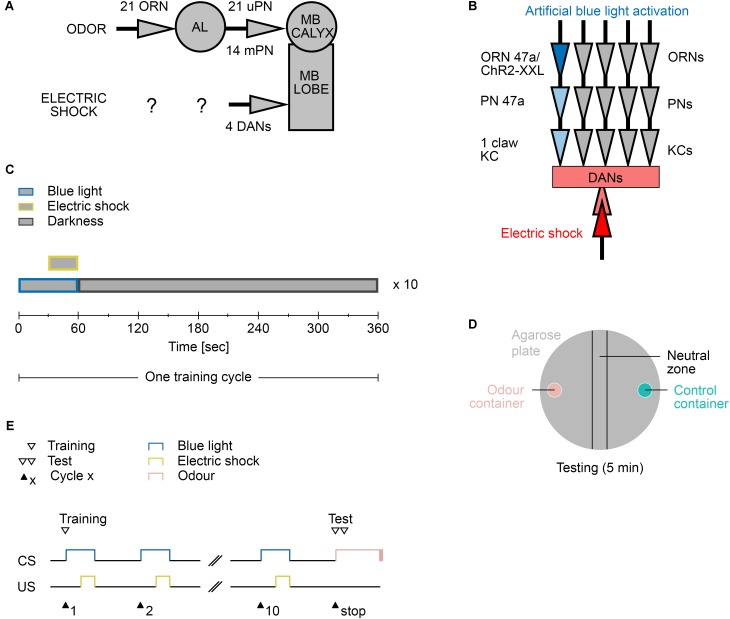
Aversive learning paradigm. **(A)** The neuronal circuit involved is depicted as the olfactory pathway (CS, on top) and the electric shock pathway (US, bottom). Olfactory information is perceived by only 21 olfactory receptor neurons (ORN) and further processed at the antennal lobe (AL). Second order projections neurons (PN) signal onto third order Kenyon Cell of the mushroom body (MB). There are 21 uni-glomerular PNs and 14 multi-glomerular PNs. Electric shocks are perceived and processed by yet unknown neurons. Further downstream likely four dopaminergic neurons (DAN) signal onto the MB lobes, where CS and US converge. **(B)** The applied protocol uses blue light activation of the single ORN 47a via Channelrhodopsin2-XXL (ChR2-XXL). Further downstream at the MB this information converges with the applied electric shock dependent activation of DANs. **(C)** Composition of one training cycle. One cycle comprises a 60 s blue light phase, in which last 30 s an electric shock is applied, and a 300 s darkness phase. The training cycle is repeated ten times. **(D)** Schematic description of the testing agarose plate. During the testing phase larvae were placed in the beginning in the neutral zone and were left on the plate for 5 min to make a decision between the presented odor (odor container; pink) and control container (empty or containing paraffin oil; turquoise). After testing, all larvae on the odor container side, the control container side, and in the neutral zone were counted. **(E)** Timescale of the larvae training and testing procedure. CS, conditioned stimulus (blue light); US, unconditioned stimulus (electric shock).

As shown in several studies, *Drosophila* larvae can establish an aversive olfactory memory by associating an odor with an electric shock (Aceves-Pina and Quinn, 1979; Heisenberg et al., 1985; Tully et al., 1994; Khurana et al., 2009; Pauls et al., 2010a). The current model suggests that the olfactory information is signaled from ORNs via PNs to MB KCs (Ramaekers et al., 2005). MB KCs, which are third-order olfactory neurons, are also stimulated via DANs, which signal a negative reinforcement (Selcho et al., 2009). When both stimuli coincide, synaptic plasticity occurs. These changes imply that in the following test, MBONs can be addressed by the learned odor to trigger the learned behavior (**Figures 2A,B**). In the standard assays, odors are used as CS. However, extensive preliminary tests have shown that using odors lead to different problems, including sticking to the agarose substrate in the training chamber (data not shown). Agarose is required to provide a substrate on which larvae can crawl easily and to prevent the larvae from drying out (Apostolopoulou et al., 2014). For this reason, we decided to train the larvae not with real odors but through the optogenetic activation of individual ORNs. Honda et al. (2014) have shown that the artificial optogenetic activation of a single ORN is sufficient to induce an associative olfactory memory in *Drosophila* larvae.

The two-odor reciprocal training paradigm is a widely used method to study associative olfactory learning and memory in larvae (Aceves-Pina and Quinn, 1979; Gerber and Stocker, 2007; Schipanski et al., 2008; Eschbach et al., 2011; von Essen et al., 2011; El-Keredy et al., 2012; Widmann et al., 2017). The use of a similar design would therefore allow for the comparison of larval odor-taste and odor-electric shock learning and memory in general. However, in an early study, we have shown that this design features several caveats (Pauls et al., 2010a). (i) The method yields relative low performance scores and thus may cause difficulty in the comparative studies of genetically manipulated larvae. (ii) This drawback may be partially overcome by increasing the number of training cycles but trigger starvation-dependent effects. (iii) The two-odor design causes a sequence effect as differences are observed in the performance depending on whether the first (CS1) or second odor (CS2) has been punished. To overcome these concerns, we decided to use exactly the same one-odor non-reciprocal training design parameters, which we have established in our previous work (Pauls et al., 2010a).

The automated training protocol consists of a 60 s blue light phase, in which an electric shock is applied during the last 30 s, followed by a 300 s resting phase in complete darkness (**Figures 2C,E**). The training trial is repeated 10 times (from now on called 10-cycle training). Immediately thereafter, the larvae are tested for 5 min for their odor preference for a specific odor over paraffin oil, which serves as the control (**Figure 2D**). The test therefore requires a manual step.

### Pairing Optogenetic Or47a Activation With Electric Shock Reduces Larval Preferences for Amyl Acetate

To demonstrate that *Drosophila* larvae can be trained in an automated fashion via the Maggot Instructor, different parameters had to be tested in advance. We used the artificial blue-light dependent activation of Or42b-Gal4 and Or47a-Gal4 crossed with UAS-ChR2-XXL to specifically activate ORN 42b and 47a, respectively (Dawydow et al., 2014; Honda et al., 2014). Both lines were reported to be single-cell-specific (Fishilevich et al., 2005). ORN 47a was reported to specifically encode the odor amyl acetate (AM), whereas ORN 42b encodes the odor ethyl acetate (EA) (Kreher et al., 2005; Hoare et al., 2011).

**FIGURE 3:**
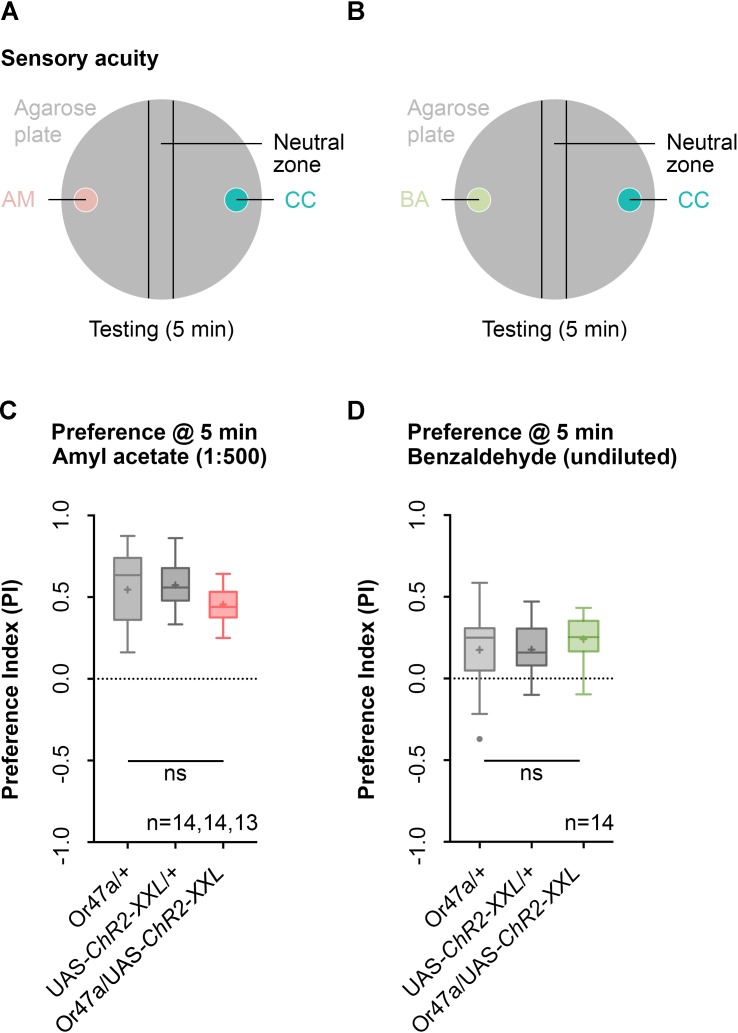
Naïve olfactory choice for amyl acetate and benzaldehyde. **(A)** Schematic representation of naïve olfactory choice for amyl acetate. Olfactory perception is analyzed by putting about 30 larvae in the middle of a Petri dish with an amyl acetate containing odor container (AM, red) on one side and an paraffin oil containing container (CC, turquoise) on the other side. After 5 min larvae are counted to calculate an olfactory preference index. **(B)** Schematic representation of naïve olfactory choice for amyl acetate. Olfactory perception is analyzed by putting 30 larvae in the middle of a Petri dish with a benzaldehyde containing odor container (BA, green) on one side and an empty container (CC, turquoise) on the other side. After 5 min larvae are counted to calculate an olfactory preference index. **(C)** The behavioral response for amyl acetate (1:500 dilution) of Or47a-Gal4/UAS-ChR2-XXL, Or47a-Gal4/+ and UAS-ChR2-XXL/+ larvae were statistically not significant from each other (Kruskal–Wallis, *p* = 0.0.118). All three groups showed an olfactory preference index statistically significantly different from zero (one sample *t*-test, *p* < 0.0001, for all three groups). **(D)** The behavioral response for benzaldehyde (undiluted) in Or47a-Gal4/UAS-ChR2-XXL, Or47a-Gal4/+ and UAS-ChR2-XXL/+ larvae were statistically not significant from each other (one-way ANOVA, *p* = 0.5757). All three groups showed an olfactory preference index statistically significantly different from zero (one sample *t*-test, *p* = 0.0196, *p* = 0.0012, *p* < 0.0001, respectively). Differences between groups are depicted below the respective box plots, at which ns indicates *p* ≥ 0.05. Small circles indicate outliers. Sample size is indicated with the letter n.

We initially focused our analysis on ORN 47a and checked whether the larvae that express ChR2-XXL in ORN 47a can perceive odors. The larvae were tested for their naïve olfactory choice behavior between an odor-filled container on one side and a container without olfactory cue on the other side of a Petri dish (**Figure 3**). This test was performed with either AM or benzaldehyde (BA) as odor stimuli (**Figures 3A,B**). Or47a-Gal4/UAS-ChR2-XXL larvae are attracted by the odor AM (**Figure 3C**). This behavioral response shows no significant difference from both the control groups (Or47-Gal4/+ and UAS-ChR2-XXL/+) (**Figure 3C**). Similarly, BA is attractive to Or47a-Gal4/UAS-ChR2-XXL larvae, and the response is comparable in both control groups (Or47-Gal4/+ and UAS-ChR2-XXL/+) (**Figure 3D**). We concluded that the expression of ChR2-XXL in ORN 47a exerts no influence on the naïve odor perception of the larvae.

**FIGURE 4:**
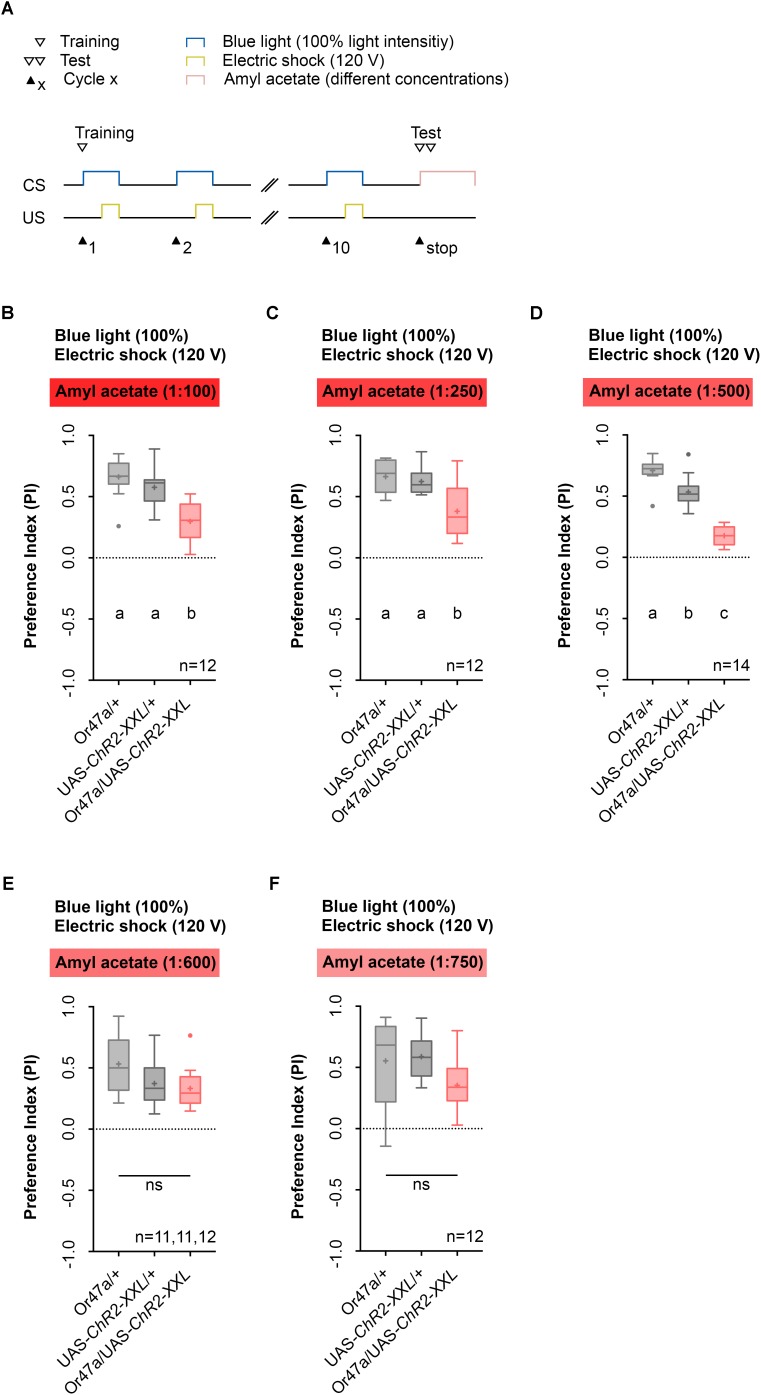
Pairing optogenetic Or47a activation with electric shock leads to the formation of odor-electric shock learning and memory in *Drosophila* larvae tested at lower amyl acetate dilutions. **(A)** Timescale of associative conditioning using 10 cycles, 120 V for electric shocks and continuous blue light with an intensity of 100%. For the olfactory preference test amyl acetate with different dilutions (1:100, 1:250, 1:500, 1:600, and 1:750) was used. **(B)** The expression of ChR2-XXL in ORN 47a led to a reduction of olfactory preference for amyl acetate at a dilution of 1:100 (Tukey *post hoc* test, *p* < 0.0001, *p* = 0.0003, respectively). All three groups showed (an olfactory preference for amyl acetate statistically significant from zero (one sample *t*-test, *p* < 0.0001 for all three groups). **(C)** The expression of ChR2-XXL in ORN 47a led to a reduction of olfactory preference for amyl acetate at a dilution of 1:250 (Dunn’s multiple pairwise comparison, *p* = 0.0.0035, *p* = 0.0307, respectively). All three groups showed olfactory preferences for amyl acetate statistically significant from zero (one sample *t-*test, *p* < 0.0001 for all three groups). **(D)** The expression of ChR2-XXL in ORN 47a led to a reduction of olfactory preference for amyl acetate at a dilution of 1:500 (one-way ANOVA, *p* < 0.0001). However, both control groups (Or47-Gal4/+ and UAS-ChR2-XXL/+) exhibited olfactory preferences, which are statistically significant form each other (Tukey *post hoc* test, *p* = 0.0001). All three groups showed an olfactory preference for amyl acetate statistically significant from zero (one sample *t*-test, *p* < 0.0001 for all three groups). **(E)** All three groups showed olfactory preferences for amyl acetate at a dilution of 1:600, which are statistically significant from zero (one sample *t*-test, *p* < 0.0001 for all three groups) but statistically not significant from each other (one-way ANOVA, *p* = 0.057). **(F)** All three groups showed olfactory preferences for amyl acetate at a dilution of 1:750, which are statistically significant from zero (one sample *t*-test, *p* = 0.0002, *p* < 0.0001, *p* = 0.0002, respectively) but statistically not significant from each other (one-way ANOVA, *p* = 0.0746). Differences between groups are depicted below the respective box plots, at which ns indicates *p* ≥ 0.05. Different lowercase letters indicate statistical significant differences at level *p* < 0.05. Small circles indicate outliers. Sample size is indicated with the letter n.

We then tested whether the activation of ORN 47a, together with an electric shock leads to a reduction in the odor preference for AM (**Figure 4**). This reduction would indicate that an aversive olfactory memory was formed. We performed five different experiments in which the light intensity and the voltage of the electric shock remained unchanged during training, but the dilution of AM in paraffin oil in the test was either 1:100, 1:250, 1:500, 1:600, or 1:750 (**Figures 4B–F**). During training via the Maggot Instructor, all larvae received the 10-cycle training as described before (**Figures 2E**, **4A**). As a result, we observed that for the dilutions 1:100, 1:250, and 1:500, the Or47a-Gal4/UAS-ChR2-XXL larvae showed a reduced olfactory preference for AM compared with both genetic control groups (Or47a-Gal4/+ and UAS-ChR2-XXL/+) (**Figures 4B–D**). No difference was observed between the three groups when the dilution of AM was 1:600 or 1:750 in the test (**Figures 4E,F**). These results suggest that associative olfactory conditioning using the Maggot Instructor is feasible, and *Drosophila* larvae are very likely able to establish an aversive odor-electric shock memory. However, the memory can only be revealed at high odor concentrations. The olfactory preference for AM for both the control groups (Or47a-Gal4/+ and UAS-ChR2-XXL/+) statistically significantly differed from each other when a dilution of 1:500 was used (**Figure 4D**). Nevertheless, we decided to continually use this odor dilution as the experimental group (Or47a-Gal4/UAS-ChR2-XXL) features a specific behavioral phenotype in comparison with both the control groups (Or47a-Gal4/+ and UAS-ChR2-XXL/+), and we have used the lowest possible odor concentration to avoid the harmful side effects.

### The Performance After Maggot Instructor Training Depends on the Applied Electric Shock and Light Intensities

**FIGURE 5:**
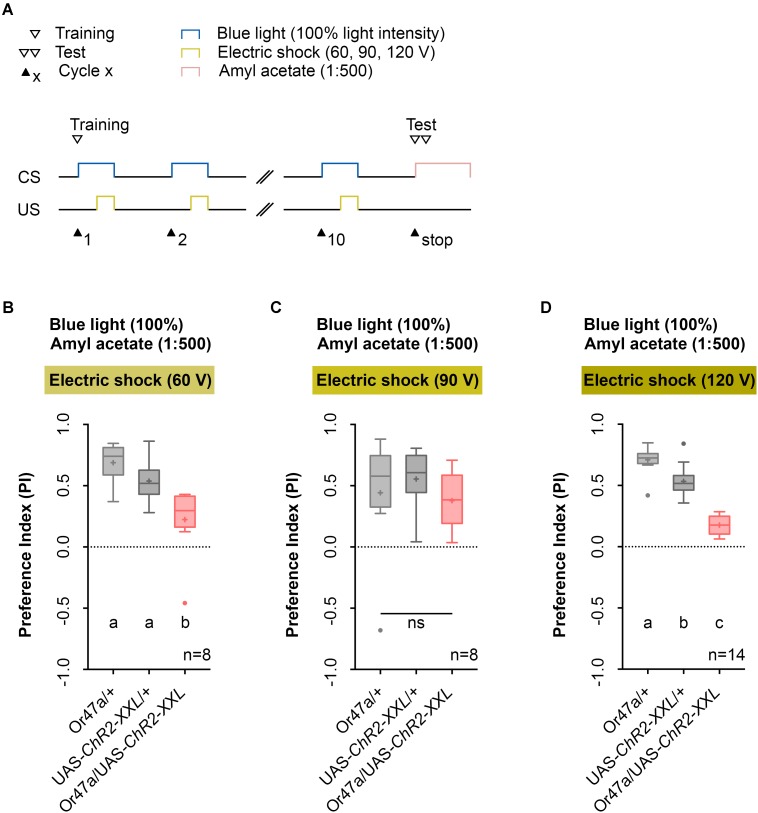
Odor-electric shock learning and memory in *Drosophila* larvae depends on the applied voltage of the electric shock. **(A)** Timescale of associative conditioning using 10 cycles, different voltages for electric shocks (60, 90, and 120 V) and continuous blue light with an intensity of 100%. For the olfactory preference test amyl acetate with a dilution of 1:500 was used. **(B)** Using 60 V in the training procedure led to a reduction of olfactory preferences for Or47-Gal4/UAS-ChR2-XXL larvae compared to both genetic controls (Or47-Gal4/+ and UAS-ChR2-XXL/+) (Tukey *post hoc* test, *p* = 0.001, *p* = 0.0168, respectively). Both genetic controls showed olfactory preferences, which are statically significant from zero (one sample *t*-test, *p* < 0.0001 for both groups), whereas Or47-Gal4/UAS-ChR2-XXL larvae showed an olfactory preference, which is not statistically significant from zero (one sample *t*-test, *p* = 0.068). **(C)** Using 90 V in the training procedure led to a reduction of olfactory preferences for all three groups, which are statistically not significant from each other (one-way ANOVA, *p* = 0.5917). All three groups showed olfactory preferences, which are statically significant from zero (one sample *t*-test, *p* = 0.0375, *p* = 0.0004, *p* = 0.0025, respectively). **(D)** The olfactory preference for amyl acetate conditioned with 120 V was already analyzed in **Figure 4D** and is just shown for comparison. Differences between groups are depicted below the respective box plots, at which ns indicates *p* ≥ 0.05. Different lowercase letters indicate statistically significant differences at level *p* < 0.05. Small circles indicate outliers. Sample size is indicated with the letter n.

**FIGURE 6:**
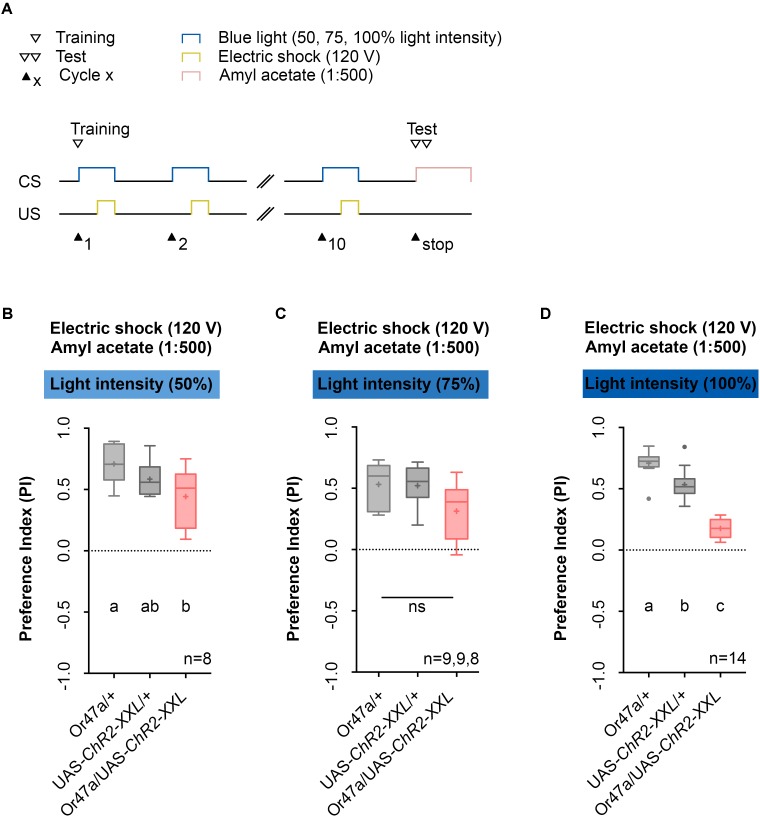
Odor-electric shock learning and memory in *Drosophila* larvae is dependent on the intensity of the blue light. **(A)** Timescale of associative conditioning using 10 cycles, 120 V for electric shocks and continuous blue light with different intensities (50, 75, and 100%). For the olfactory preference test amyl acetate with a dilution of 1:500 was used. **(B)** Using a light intensity of 50% in the training procedure led to olfactory preferences, which are statistically significant within the three groups (one-way ANOVA, *p* = 0.0288). However, the difference was only statistically significant between Or47-Gal4/UAS-ChR2-XXL and Or47-Gal4/+ larvae (Tukey *post hoc* test, *p* = 0.0222), whereas the olfactory preferences for Or47-Gal4/UAS-ChR2-XXL and UAS-ChR2-XXL/+ larvae were not statistically significant from each other (Tukey *post hoc* test, *p* = 0.2906). **(C)** Using a light intensity of 75% in the training procedure led to olfactory preferences, which are statistically not significant from each other (one-way ANOVA, *p* = 0.0522). All three groups showed olfactory preferences, which are statically significant from zero (one sample *t*-test, *p* < 0.0001, *p* < 0.0001, *p* = 0.007, respectively). **(D)** The olfactory preference for amyl acetate conditioned with 120 V was already analyzed in **Figure 4D** and is just shown for comparison. Differences between groups are depicted below the respective box plots, at which ns indicates *p* ≥ 0.05. Different lowercase letters indicate statistically significant differences at level *p* < 0.05. Small circles indicate outliers. Sample size is indicated with the letter n.

Next, we performed a parametric analysis with varying voltage of the applied electric shock and intensity of the artificial blue light activation (**Figures 5**, **6**). We used the established 1:500 AM dilution and the 10-cycle protocol (**Figure 5A**) and tested whether electric shocks applied at 60, 90, or 120 V cause different effects on learning and memory (**Figures 5B–D**). As a result, we noted that for electric shocks of 60 and 120 V, in contrast to 90 V, Or47a-Gal4/UAS-ChR2-XXL larvae showed a reduced olfactory preference for AM compared with both the genetic control groups (Or47a-Gal4/+ and UAS-ChR2-XXL/+) (**Figures 5B–D**). Based on this results, we continually used 120 V for electric shocks, as all larvae survived this treatment and showed slightly stronger differences between the experimental group and both controls.

Next, we used the 1:500 AM dilution, 10-cycle, and 120 V protocol (**Figure 6A**) to test whether three different blue light intensities (50%, 75%, or 100%) cause different effects on learning and memory (**Figures 6B–D**). We noted that for blue light intensities of 100%, Or47a-Gal4/UAS-ChR2-XXL experimental larvae showed a reduced olfactory preference for AM compared with both genetic controls (Or47a-Gal4/+ and UAS-ChR2-XXL/+) (**Figure 6D**). By contrast, when trained with blue light intensities of 50% and 75%, the Or47a-Gal4/UAS-ChR2-XXL larvae showed no significant reduction in their preference for AM compared with both or at least one genetic control (**Figures 6B,C**; for blue light intensities of 50%, a significant difference was observed between Or47a-Gal4/+ and Or47a-Gal4/ChR2-XXL). Based on this result, we used a blue light intensity of 100% for follow-up experiments.

### Lithium Chloride Application or Pulsed Blue-Light Causes no Improvement in the Training Protocol

**FIGURE 7:**
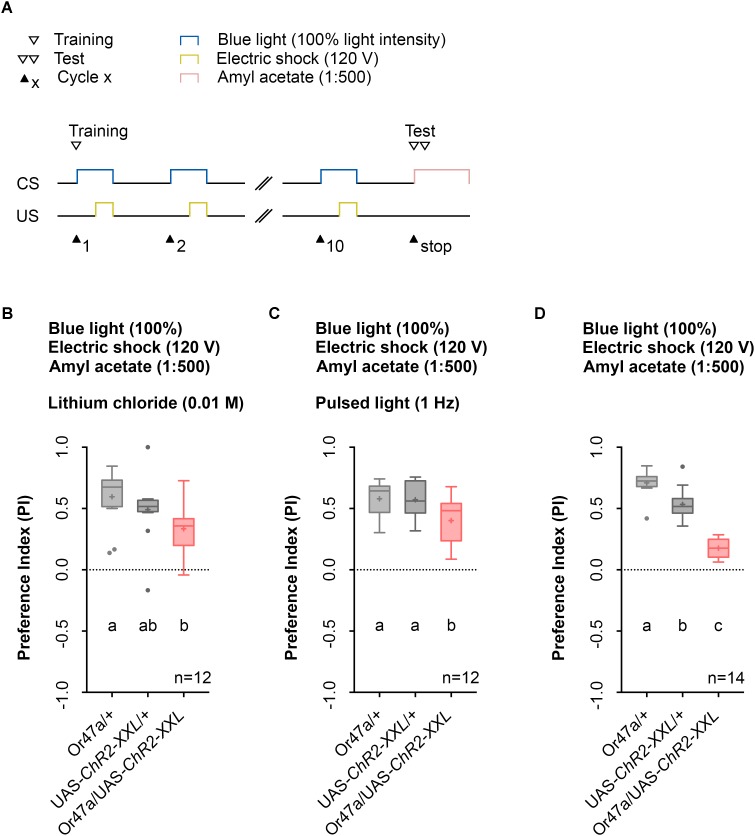
The usage of lithium chloride (LiCl) or pulsed light does not have a significant effect of odor-electric shock learning and memory. **(A)** Timescale of associative conditioning using 10 cycles, 120 V for electric shocks and continuous blue light with an intensity of 100%. For the olfactory preference test amyl acetate with a dilution of 1:500 was used. **(B)** Mixing LiCl at a concentration of 0.01 M into agarose led to a olfactory preference for amyl acetate, which differs statistically significant between Or47-Gal4/+ and Or47-Gal4/UAS-ChR2-XXL larvae (Dunn’s multiple comparison, *p* = 0.0094) but not between UAS-ChR2-XXL/+ and Or47-Gal4/UAS-ChR2-XXL larvae (Dunn’s multiple comparison, *p* = 0.2697) and both control groups (Dunn’s multiple comparison, *p* = 0.6232). All three groups showed olfactory preferences for amyl acetate statistically significant from zero (Wilcoxon signed rank test, *p* = 0.0005, *p* = 0.001, *p* = 0.001, respectively). **(C)** Using pulsed blue light with an intensity of 100% for the optogenetic activation of Or47a led to olfactory preference for Or47-Gal4/UAS-ChR2-XXL larvae, which is statistically significant to both control groups (Tukey *post hoc* test, *p* = 0.0254, *p* = 0.0346, respectively). All three groups showed olfactory preferences, which are statistically significant from zero (one sample *t*-test, *p* < 0.0001 for all three groups). **(D)** The olfactory preference for amyl acetate conditioned with a continuous blue light intensity of 100% and without adding LiCl was already analyzed in **Figure 4D** and is shown for comparison. Differences between groups are depicted below the respective box plots, at which ns indicates *p* ≥ 0.05. Different lowercase letters indicate statistical significant differences at level *p* < 0.05. Small circles indicate outliers. Sample size is indicated with the letter n.

Previous studies that used LiCl reported an increase in larval memory scores for odor-electric shock learning as it makes the agarose substrate electrically conductive while being tasteless for larvae (Aceves-Pina and Quinn, 1979). However, this effect could not be confirmed by a study from our laboratory (Pauls et al., 2010a). Nonetheless, we determined whether the use of LiCl affects the automated Maggot Instructor training as its intake might cause harmful effects for the larvae and was reported to modulate adult behavior (Ries et al., 2017). The obtained data revealed that the use of LiCl is not necessary in our setup (**Figure 7B**), similar to our published data (Pauls et al., 2010a).

Prolonged blue-light activation of the sensory neurons via ChR2-XXL can lead to a decrease in firing of the cells (Dawydow et al., 2014). Therefore, we tested whether pulsed blue light activation of ORN 47a may produce a stronger behavioral effect. Instead, of a constant blue light activation of 60 s we used an alternating 1 s on-off regime. In this case, Or47a-Gal4/UAS-ChR2-XXL experimental larvae showed a significant reduction in their odor preference compared with the Or47a-Gal4/+ and UAS-ChR2-XXL/+ control groups (**Figure 7C**). Direct comparison of the performance of Or47a-Gal4/UAS-ChR2-XXL larvae at pulsed light (**Figure 7C**) and constant light (**Figure 7D**) showed a significant difference in the odor preference between both groups. This result indicates that the optogenetic activation with pulsed light featured a weaker effect on reducing odor preferences for AM than with constant light. Therefore, we continually used the 1:500 AM dilution, 10-cycle, 120 V, and 100% constant blue light protocol on the agarose filled training chambers without LiCl.

### Additional Control Experiments Support the Associative Nature of the Learning and Memory Phenotype

**FIGURE 8:**
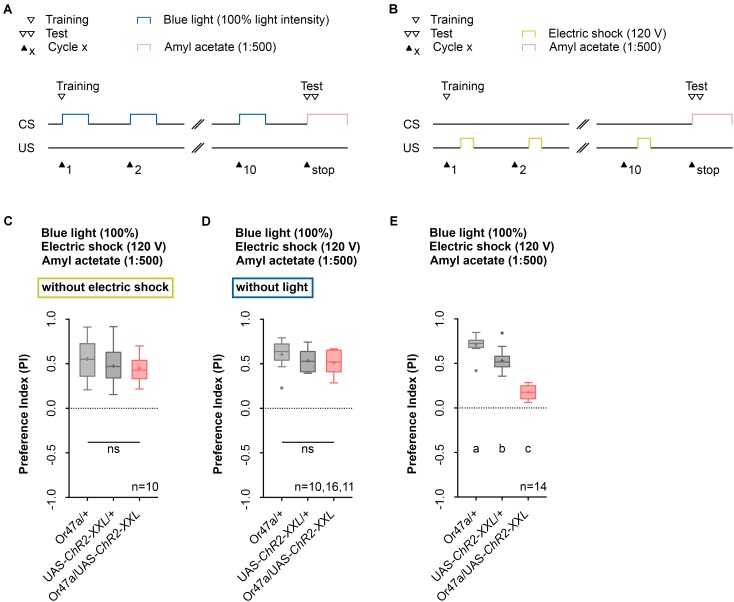
Odor-electric shock learning and memory depends on the simultaneous blue light activation and electric shock stimulation. **(A)** Timescale of associative conditioning using 10 cycles, continuous blue light with an intensity of 100%, without electric shock. For the olfactory preference test amyl acetate with a dilution of 1:500 was used. **(B)** Timescale of associative conditioning using 10 cycles, 120 V for electric shocks, but without continuous blue light. For the olfactory preference test amyl acetate with a dilution of 1:500 was used. **(C)** Associative conditioning without electric shock stimulation but optogenetic Or47a activation led to olfactory preferences, which are statistically not significant within the three groups (one-way ANOVA, *p* = 0.4062). All three groups showed olfactory preferences, which are statistically significant from zero (one sample *t*-test, *p* < 0.0001 for all three groups). **(D)** Associative conditioning without optogenetic Or47a activation but electric shock stimulation led to olfactory preferences, which are statistically not significant within the three groups (one-way ANOVA, *p* = 0.3355). All three groups showed olfactory preferences, which are statistically significant from zero (one sample *t*-test, *p* < 0.0001 for all three groups). **(E)** The olfactory preference for amyl acetate conditioned with 120 V was already analyzed in **Figure 4D** and is just shown for comparison. Differences between groups are depicted below the respective box plots, at which ns indicates *p* ≥ 0.05. Different lowercase letters indicate statistical significant differences at level *p* < 0.05. Small circles indicate outliers. Sample size is indicated with the letter n.

The conditioning regime used by the Maggot Instructor lacks reciprocity. The regime defines learning and memory as a reduction in AM preference between an experimental group and two genetic control groups. We thus designed two additional control experiments to ensure that neither blue light activation nor electric shock stimulation alone specifically can change the AM preference of Or47a-Gal4/UAS-ChR2-XXL larvae (**Figures 8A,B**). Although unlikely, significant differences between the experimental and control groups would suggest that the obtained phenotype would be based on non-associative effects rather than associative learning and memory. As expected, both results showed no reduction in the AM preference of the Or47a-Gal4/UAS-ChR2-XXL larvae compared with the Or47a-Gal4/+ and UAS-ChR2-XXL/+ control groups (**Figures 8C,D**). These results show that the observed behavioral change in the experimental larvae after conditioning via the Maggot Instructor is based on associative learning and memory.

### Artificial Activation of Distinct ORNs Establishes Odor-Specific Memories

**FIGURE 9:**
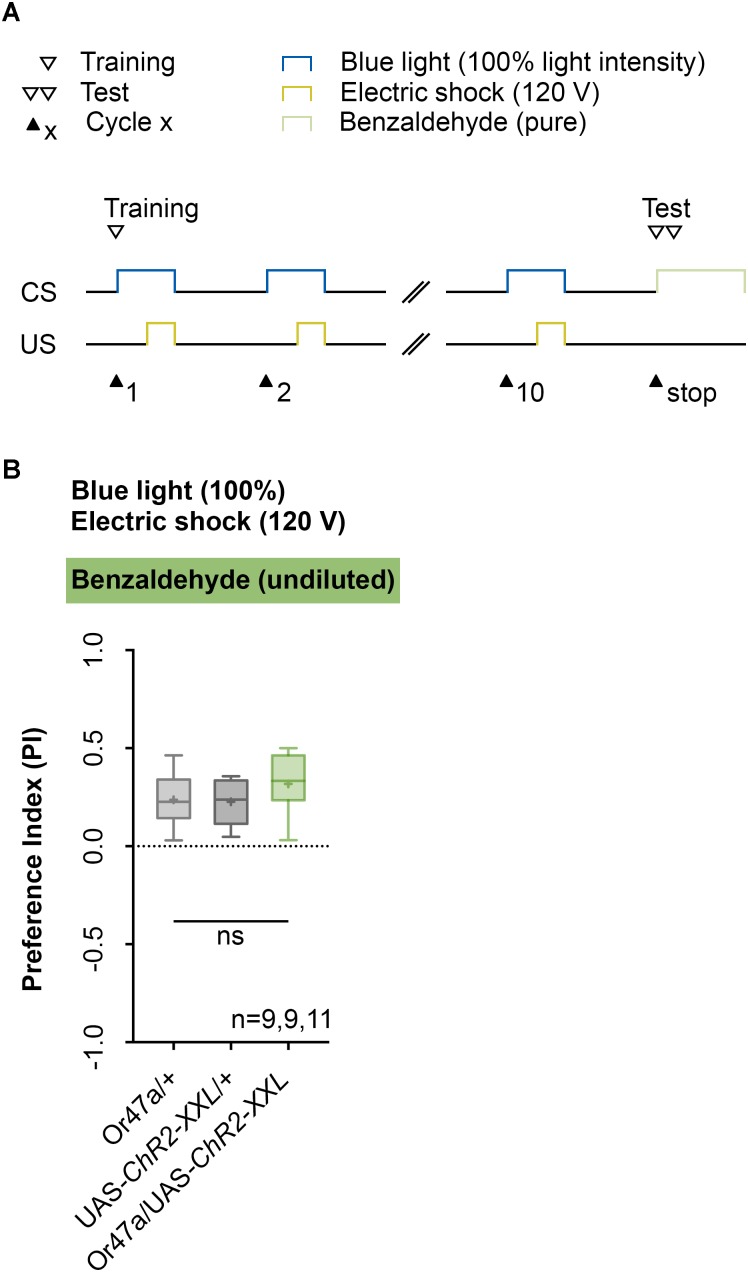
Artificial activation of ORN 47a establishes an odor-electric shock memory, which is specific for amyl acetate. **(A)** Timescale of associative conditioning using 10 cycles, 120 V for electric shocks and continuous blue light with an intensity of 100%. For the olfactory preference test benzaldehyde (undiluted) was used. **(B)** Using benzaldehyde in the test led to olfactory preferences, which are statistically not significant within the three groups (one-way ANOVA, *p* = 0.254). All three groups showed olfactory preferences, which are statistically significant from zero (one sample *t*-test, *p* = 0.0007, *p* = 0.0004, *p* < 0.0001, respectively). Differences between groups are depicted below the respective box plots, at which ns indicates *p* ≥ 0.05. Different lowercase letters indicate statistical significant differences at level *p* < 0.05. Small circles indicate outliers. Sample size is indicated with the letter n.

**FIGURE 10:**
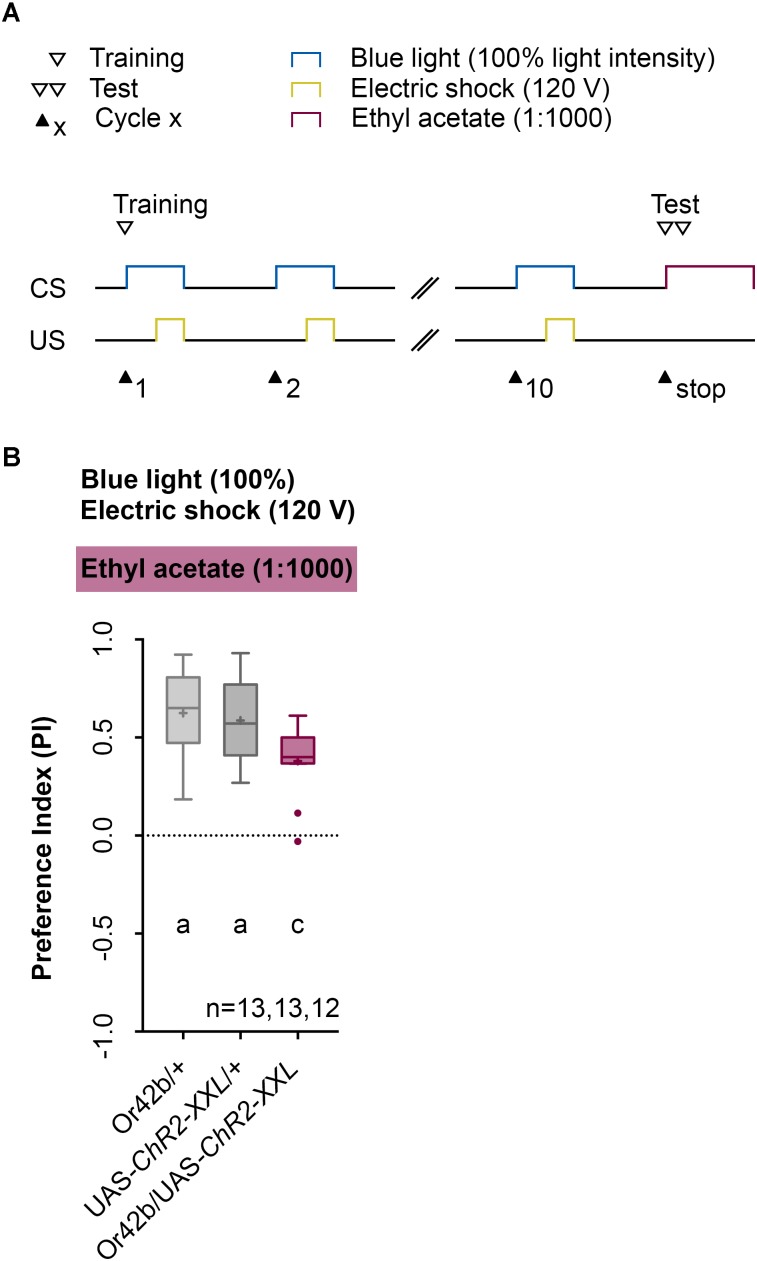
Establishing an odor-electric shock memory through artificial activation of specific ORNs and simultaneous stimulation is a general property of *Drosophila* larvae. **(A)** Timescale of associative conditioning using 10 cycles, 120 V for electric shocks and continuous blue light with an intensity of 100%. For the olfactory preference test ethyl acetate (1:1000) was used. Here, continuous blue light activates Or42b, which has a reported response profile for ethyl acetate. **(B)** The artificial activation of Or42b and using ethyl acetate in the test led to olfactory preferences for Or42b/UAS-ChR2-XXL, which are statistically significant to both control groups (Or42b/+ and UAS-ChR2-XXL/+) (Tukey *post hoc* test, *p* = 0.0133, *p* = 0.0398, respectively). All three groups showed olfactory preferences, which are statistically significant from zero (one sample *t*-test, *p* < 0.0001, for all three groups). Differences between groups are depicted below the respective box plots, at which ns indicates *p* ≥ 0.05. Different lowercase letters indicate statistical significant differences at level *p* < 0.05. Small circles indicate outliers. Sample size is indicated with the letter n.

Next, we analyzed the odor specificity of the memory. Studies previously showed that artificial activation of a ORN during conditioning induces an odor-specific memory that overlaps with the response profile predicted for the respective ORN (Honda et al., 2014). Accordingly, we tested whether the artificial activation of ORN 47a can also establish odor-electric shock learning and memory for an odor that is not covered by the reported Or47a response profile. Considering Or47a, such case applies to BA (Kreher et al., 2005; Hoare et al., 2011; Munch and Galizia, 2016). As expected Or47a-Gal4/UAS-ChR2-XXL larvae showed an odor preference for BA, and this preference is indistinguishable from the both genetic control groups (Or47a-Gal4/+ and UAS-ChR2-XXL/+) (**Figure 9B**). Based on this result we conclude that odor-electric shock learning and memory established after training via the Maggot Instructor is specific for the activated ORN and thus overlaps with its reported response profile. We confirmed this result independently by reproducing the finding published for Or42b. Honda et al. (2014) reported that the artificial activation of ORN 42b paired with an artificial activation of octopaminergic neurons that encode for a rewarding function establishes an appetitive olfactory memory specific for EA. Using our standardized training protocol but the odor EA (1:1000) in the test (**Figure 10A**) Or42b-Gal4/UAS-ChR2-XXL larvae also established an aversive odor-electric shock memory (**Figure 10B**).

## Discussion

### The Maggot Instructor Trains Larvae in an Automated Fashion to Establish an Associative Olfactory Memory

*Drosophila* larvae can establish different types of associative memory based on the pairing of two stimuli (US and CS) (Aceves-Pina and Quinn, 1979; Scherer et al., 2003; Gerber and Stocker, 2007; Widmann et al., 2017). In contrast to the almost exclusively manual assays that are currently in use, we showed that larvae can also be trained automatically with the help of the Maggot Instructor. Automation will allow one to conduct comprehensive behavioral screens of newly established genetic tools (Li et al., 2014; Saumweber et al., 2018). In several experiments, we have shown that genetically modified larvae, which still show a natural naïve odor preference (**Figure 3**), learn the temporal paired optogenetic activation of ORN 47a with an electric shock and store this experience as an aversive olfactory memory (**Figures 4**–**7**, **10**). Our results showed that this memory is specific for the identity and concentration of odors as the odor-electric shock memory was only detectable at certain concentrations of AM (**Figure 4**) and not visible when BA was used in the test (**Figure 9**). The conclusion regarding the associative nature of the observed reduction in the AM preference is compelling as we also showed that other parameters *per se*, such as artificial activation and electric shock, caused no alteration in the tested olfactory behavior (**Figure 8**). Therefore, we conclude that training larvae via the Maggot Instructor leads to an odor-specific associative process. The formation of memory by artificial activation of ORNs is not limited to ORN 47a given that an EA memory can be formed through the activation of ORNS 42b (**Figure 10**). However, for each of the 21 ORNs, odor-specific associative processes have to be tested, as several studies have shown the presence of non-equivalency among larval ORNs (Mathew et al., 2013; Hernandez-Nunez et al., 2015; Newquist et al., 2016). ORN 42a, for instance, unlike many other larval ORNs was shown to respond to a wide range of odors (Kreher et al., 2005; Hoare et al., 2011; Mathew et al., 2013).

### Real World Stimulation or Artificial Activation of Distinct Neurons of the Learning and Memory Network

To establish an associative olfactory memory in *Drosophila* larvae, the animals with natural stimuli, such as an odor and an electric shock, must be conditioned (Aceves-Pina and Quinn, 1979; Pauls et al., 2010a). However, the precise control of natural stimuli often presents difficulty. Therefore, thermogenetic and optogenetic effectors, such as TRPA1 and ChR2, that are expressed via transgenic techniques provide an alternative as they allow for the precise control of the activity of defined neurons in living larvae (Hamada et al., 2008; Dawydow et al., 2014). Associative olfactory conditioning theoretically includes the CS (odor) and/or the US (reward/punishment) pathways. Schroll et al. (2006) showed that light-induced activation of a set of DA neurons paired with an odor stimulus induces aversive memory formation, whereas activation of OA neurons induces appetitive memory formation. These results could be extended by demonstrating that in downstream of the OA neurons, the activity of four DA pPAM is also sufficient to trigger an appetitive memory (Rohwedder et al., 2016). For two of these DA neurons, activating them individually is enough for memory formation (Saumweber et al., 2018). In summary, these studies showed that substation experiments can be possibly carried out for the US in the larva, both for appetitive and for aversive learning, up to the single-cell level. This condition also holds true for the adult *Drosophila*. By contrast, a successful CS substitution at the level of ORN has thus far only been shown for the larva stage (Honda et al., 2014). Perhaps, the reason is the simpler neural network or the organization of parts of the larval olfactory pathway as a labeled line up to the MB (Ramaekers et al., 2005; Berck et al., 2016; Eichler et al., 2017). The optogenetic activation of ORN 24a and ORN 42b paired with the thermogenetic activation of most OA neurons induces an appetitive memory for acetophenone and EA, respectively (Honda et al., 2014). In this study, we showed for the first time the establishment of an aversive memory via CS substitution (**Figure 10**). Taken together the activation of ORN 42b serves the classical CS function. The pairing of ORN 42b activation via a natural odor or artificially via blue light and a reward or punishment causes the CS to trigger attraction or avoidance. As a consequence, appetitive and aversive associative learning processes can now be generated artificially, temporally, and spatially in various combinations in the larval brain and independent of natural stimuli. In this situation, the Maggot Instructor can be helpful. Thus, in future experiments, the order of CS and US, their precise timing (e.g., backward and forward conditioning; delay conditioning), and additional parameters, such as the number of training cycles or the strength of the CS and the US, can be analyzed in a controlled manner. The same condition applies to the neuronal networks. Activation experiments for PNs, sets of KCs, MBONs, and screens for identifying neurons of the US pathway would be conceivable.

### Meaning of the Artificial ORN Activation

The associative olfactory learning and memory that we tested with ORN 47a was specific for AM (**Figures 4–9**). However, we opted not to analyze in-depth the odor specificity of the memory. The tuning curve for the receptor Or47a is very specific at low odor concentrations (10^-4^) and responded almost exclusively to AM when tested for 26 different odors (Kreher et al., 2008). This result was also confirmed by a second study, which has tested for 19 different odors (Hoare et al., 2011). We used these results to select Or47a for our experiments. At a higher concentration (10^-2^), the receptor specificity changes, and in addition to AM, one also sees responses to other odors, such as propyl acetate, isoamyl acetate, 1-octen-3-ol, and 2-heptanone. For the receptor Or42b, this condition is very similar. At low concentrations (10^-4^) Or42b shows high specificity for EA. At high concentrations (10^-2^) responses for ethyl butyrate, propyl acetate, 2,3-butanedione and potential AM are reported (Kreher et al., 2008; Hoare et al., 2011). The high throughput rate of the Maggot Instructor allows repetition of these physiological experiments at the behavioral level to identify the tuning curves for each ORN in relation to many odors after olfactory learning and memory. These experiments would provide more information on the neural principles of larval odor processing to better understand the odors that larvae can learn and remember.

### Technical Caveats

The Maggot Instructor shortens the time necessary to perform an experiment. The manual training protocol consists of 60 s CS and US pairing followed by a 300 s resting phase in complete darkness (**Figures 2C,E**). This training trial is repeated 10 times and spans 60 min in total (Pauls et al., 2010a). Although the Maggot Instructor, compared with the manual protocol, requires about the same time to prepare the larvae before and test them after training, the training itself requires no handling. A standard experiment usually consists of an experimental group, a driver and reporter control, each with about 10 repetitions per genotype. This situation results in a time of approximately 3 (genotypes) × 10 (repetitions) × 60 min, or 30 h saved per complete experiment.

Although this rough estimate shows the immense time saved, one must also mention that large genetic screens cannot be achieved immediately. The Maggot Instructor requires ChR2 to be expressed in individual ORNs. This goal can be achieved either via direct Or promoter ChR2 fusion constructs, via the LexA, the Q, or the Gal4/UAS system (Brand and Perrimon, 1993; Lai and Lee, 2006; Potter et al., 2010). However, as these tools are either non-existent, rare, or problematic, and as they affect other genetic modifications, genetic screens require a special strategy to deploy the Maggot Instructor. For example establishing the Or47::ChR2-XXL larvae would be possible. This construct can either be combined with a MB-Gal4 line to screen for the requirement of individual genes using available UAS-RNAi lines or with UAS-shi^ts^ to use available Gal4 and split-Gal4 lines to identify the neuronal circuits and individual neurons required for learning and memory (Kitamoto, 2001; Pfeiffer et al., 2010; Li et al., 2014). Alternatively, one can combine Or-LexA with LexAop-ChR2-XXL to artificially activate individual ORNs (Selcho et al., 2017). However, to date, to our knowledge, only Or47b-LexA (Hueston et al., 2016), which is not expressed in the larval olfactory system, has been published; thus, one would have to establish in any case new genetic tools before one can use the Maggot Instructor for large genetic screens.

### Outlook

In this work, we exclusively focused on the aversive olfactory memory reinforced with electric shock. The design of the Maggot Instructor, however, allows a whole series of other applications. *Drosophila* larvae can also associate odor information with light or heat punishment (von Essen et al., 2011; Khurana et al., 2012). The Maggot Instructor can apply these stimuli automatically. Furthermore, the Maggot Instructor offers the possibility to analyze associative visual learning and memory by pairing a light stimulus with electric shock. Such a protocol is already established as a manual assay (von Essen et al., 2011).

Extensive double activation experiments are also now possible. Defined ORN activation (standardized CS) can then be paired with activation of individual sensory neurons expressing gustatory receptors, ionotropic receptors, transient receptor potential cation channels, and/or pickpocket ion channel genes (Clyne, 2000; Dunipace et al., 2001; Scott et al., 2001; Liu et al., 2003; Montell, 2005; Benton et al., 2009). In this manner, one could comprehensively identify the sensory neurons that encode for appetitive and aversive reinforcement in *Drosophila* larvae (e.g., Gr93a for aversive reinforcement and IR60c potentially for appetitive reinforcement) (Apostolopoulou et al., 2016; Croset et al., 2016).

In summary, the range of applications of the Maggot Instructors extends well beyond the one shown here. Therefore, we confidently present in this work a very useful device that allows more rapid analysis of the behavioral, neuronal, and molecular fundamentals and different forms of larval learning and memory in the future.

## Author Contributions

UT conceived the study, coordinated and contributed behavioral experiments, analyzed behavioral data, designed figures, and wrote the manuscript. AW conceived the study, coordinated behavioral experiments, analyzed behavioral data, designed figures, and wrote the manuscript. AT conceived of and coordinated study, analyzed the data, designed figures, and wrote the manuscript.

## Conflict of Interest Statement

The authors declare that the research was conducted in the absence of any commercial or financial relationships that could be construed as a potential conflict of interest.
